# The potential of *Gynostemma pentaphyllum* in the treatment of hyperlipidemia and its interaction with the LOX1‐PI3K‐AKT‐eNOS pathway

**DOI:** 10.1002/fsn3.4250

**Published:** 2024-08-20

**Authors:** Zhuyang Shen, Xin Gao, Dan Huang, Xiaojin Xu, Jianping Shen

**Affiliations:** ^1^ Affiliated Hospital of Integrated Traditional Chinese and Western Medicine Nanjing University of Chinese Nanjing China; ^2^ Jiangsu Province Academy of Traditional Chinese Medicine Nanjing China

**Keywords:** active ingredients, *Gynostemma pentaphyllum*, hyperlipidemia, LOX1‐PI3K‐AKT‐eNOS, network pharmacology, protein–protein interaction

## Abstract

*Gynostemma pentaphyllum*, a traditional Chinese medicine, is widely used to treat various diseases, but its therapeutic effects and mechanisms of action on hyperlipidemia remain unclear. This study aims to investigate the effects of Danshen leaf on hyperlipidemia through network pharmacology, molecular docking, and cellular experiments, elucidating its multifaceted mechanism of action within the LOX1‐PI3K‐AKT‐eNOS pathway. First, the active ingredients and targets of *G. pentaphyllum* were screened using the Traditional Chinese Medicine Systems Pharmacology database. Then, targets for hyperlipidemia were identified using the OMIM and GeneCards databases, and potential therapeutic targets for *G. pentaphyllum* in treating hyperlipidemia were determined. An active ingredient‐target network was constructed using Cytoscape software, and a protein–protein interaction (PPI) network was built and visualized using the STRING database and Cytoscape software. Finally, GO functional and KEGG pathway enrichment analyses were performed, and the predicted mechanisms were validated through molecular docking and cell experiments. 85 targets for *G. pentaphyllum* and 1556 for Hyperlipidemia were screened, with 53 common targets. Twenty‐four active ingredients of *G. pentaphyllum* were found to be involved in the treatment of hyperlipidemia. Key nodes such as Rhamnazin, Isofucosterol, and quercetin, and targets NCOA2, NR3C2, PGR, and PPARG showed high relevance. In the PPI network, 8 nodes, including IL6, PPARG, and VEGFA, exhibited high centrality. GO functional and KEGG pathway enrichment analyses indicated that *G. pentaphyllum* may treat hyperlipidemia by influencing various biological functions and pathways, such as DNA‐binding transcription factor binding, RNA polymerase II‐specific DNA‐binding transcription factor binding, and lipid and atherosclerosis. Cell experiments demonstrated that *G. pentaphyllum* significantly regulated the expression of key proteins in the LOX1‐PI3K‐AKT‐eNOS pathway, thereby improving hyperlipidemia. *G. pentaphyllum* improves hyperlipidemia by mediating the LOX1‐PI3K‐AKT‐eNOS pathway. This study provides a new theoretical basis and experimental evidence for applying *G. pentaphyllum* to treating hyperlipidemia.

## Graphical abstract

The first documentation of *Gynostemma pentaphyllum*'s impact on the LOX1‐PI3K‐AKT‐eNOS pathway. Unveiled 24 active ingredients in *G. pentaphyllum* targeting hyperlipidemia. Highlighted rhamnazin, isofucosterol, and quercetin's crucial role in treatment.

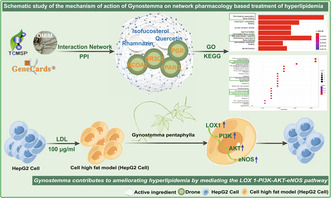

## 1 INTRODUCTION

*Gynostemma pentaphyllum*, a traditional Chinese medicine with a long history, has been widely utilized (Ji et al., 2018; Li et al., 2016, 2019). *G. pentaphyllum* has been employed throughout the ages to treat various ailments such as rheumatism, liver disease, and diabetes. Ancient medical literature provides detailed accounts of the pharmacological effects and applications of *G. pentaphyllum*. With the advancement of modern technology, numerous studies have begun exploring the bioactive components and scientific basis of its therapeutic effects. *G. pentaphyllum*, a herbaceous plant of the Cucurbitaceae family, has been extensively used in traditional Chinese medicine and contains active ingredients such as saponins, flavonoids, polysaccharides, and amino acids (Xie et al., 2024). Pharmacological research indicates that *G. pentaphyllum* exhibits a broad range of pharmacological effects, including anti‐inflammatory, anti‐tumor, lipid‐lowering, hepatoprotective activities, and cardiovascular protection (Ji et al., 2018).

Hyperlipidemia is a disease associated with lipid metabolism disorders, characterized by an increase in the concentration of cholesterol, triglycerides, or both in plasma (Jia et al., 2021; Stewart et al., 2020; Su et al., 2022). This metabolic abnormality can lead to various cardiovascular diseases, such as atherosclerosis and coronary heart disease (Libby et al., 2019). In contemporary times, with the changes in people's lifestyle habits, the incidence of hyperlipidemia has gradually increased, becoming one of the major threats to global health. Currently, drugs used in the treatment of hyperlipidemia mainly include statins, fibrates, and fish oil preparations, but these drugs may have certain side effects (He & Ye, 2020; Jia et al., 2021; Miao et al., 2020). Chinese herbal medicine is characterized by its low toxic side effects, diverse targets, and significant effects in regulating hyperlipidemia. Existing studies have reported that *G. pentaphyllum* shows promising results in improving glucose and lipid metabolism (Xie et al., 2023). Biochemical analysis has revealed that the triterpenes isolated from *G. pentaphyllum* have a cholesterol‐lowering effect (Li et al., 2023). However, the precise molecular mechanism of *G. pentaphyllum* in treating hyperlipidemia remains unclear.

Network pharmacology is an emerging interdisciplinary field that combines principles from bioinformatics, systems biology, and pharmacology, aiming to explore the systematic mechanisms of drug action (Jiashuo et al., 2022; Li et al., 2022; Nogales et al., 2022). Through network pharmacology, researchers can gain deeper insights into how drugs affect the molecular, cellular, and tissue levels, thus guiding drug design and optimization. Network pharmacology has been widely used in new drug discovery, drug repurposing, and modernizing traditional Chinese medicine research in recent years.

Based on the above research background, the treatment and prevention of hyperlipidemia by *G. pentaphyllum* are of significant importance, but their molecular mechanisms require further exploration. This study, leveraging network pharmacology, explores the potential mechanisms of *G. pentaphyllum* in treating hyperlipidemia and validates relevant molecular mechanisms through cellular experiments. We hope this study will not only provide a new theoretical foundation and experimental evidence for applying *G. pentaphyllum* in the treatment of hyperlipidemia, but also serve as a valuable reference for developing new drugs for hyperlipidemia.

## 2 MATERIALS AND METHODS

### 2.1 Screening of active compounds and corresponding targets of *G. pentaphyllum*

In this study, we initially conducted a data retrieval using the Traditional Chinese Medicine Systems Pharmacology (TCMSP) database (version: 2.3) with “*Gynostemma pentaphyllum*” as a keyword. The retrieved results provided information on the compounds and their pharmacological activity. To ensure the reliability of the data, compounds with pharmacological activity were selected from *G. pentaphyllum* based on the criteria of oral bioavailability (OB) ≥30% and drug‐likeness (DL) ≥0.18. Next, the target names of *G. pentaphyllum* obtained from the TCMSP database were standardized using the UniProt database (http://www.uniprot.org). To identify the associated targets of hyperlipidemia, we utilized the Online Mendelian Inheritance in Man (OMIM) (https://omim.org/) and GeneCards databases (https://www.genecards.org/) to extract relevant target information. By integrating these datasets, we performed an intersection analysis using the R software (version 4.2.1, https://www.r‐project.org/) to identify potential therapeutic targets of *G. pentaphyllum* in treating hyperlipidemia (Gao et al., 2022; Li et al., 2021).

### 2.2 Construction of an active component‐target of action network

To investigate the interaction between active components of *G. pentaphyllum* and their target molecules, we constructed an active component‐target network using Cytoscape software (version 3.9.1, https://cytoscape.org/). Firstly, we imported the active components of *G. pentaphyllum* and the related target data determined in section 1.1.1 into the software. In the network, each node represents an active component or target, while edges represent the interaction between active components and targets. We optimized the visualization of the network by adjusting the size, color, and shape of the nodes and edges to make it more intuitive and clear. In addition, we performed topological analysis using the network analysis tools built into Cytoscape to identify the core nodes and key connections in the network. This analysis provided valuable information for further mechanism studies and drug development (Xu et al., 2021).

### 2.3 Construction of a protein–protein interaction (PPI) network and screening of key targets

To investigate the key targets and their interactions with *G. pentaphyllum* in the treatment of hyperlipidemia, we constructed a PPI network. Firstly, we inputted the relevant targets of *G. pentaphyllum* and hyperlipidemia into the STRING database (https://string‐db.org/) for querying. In the query parameters, we selected Homo sapiens as the target species, hid the disconnected nodes in the network, and set the interaction score to the highest confidence (0.900) to ensure the reliability of the results while keeping the remaining parameters at their default settings. With these settings, we ensured that the obtained PPI network had high credibility and relevance. Next, the PPI network data from the STRING database were imported into the Cytoscape software (version 3.9.1) for network visualization and processing. To better understand the status and role of each protein in the PPI network, we performed a topological structure analysis of the PPI network using R language (version 4.2.1) and plotted enrichment maps of the proteins in the network, thereby identifying the key targets and their interaction relationships in the network, which provided strong support for subsequent mechanistic studies (Duan et al., 2021).

### 2.4 GO function and KEGG pathway enrichment analysis

To further elucidate the biological functions of *G. pentaphyllum* and its potential mechanisms in treating hyperlipidemia, we performed enrichment analysis of Gene Ontology (GO) functions and Kyoto Encyclopedia of Genes and Genomes (KEGG) pathways. Firstly, the encoded genes of relevant targets were inputted into the open‐source bioinformatics software Bioconductor (http://www.bioconductor.org/). In the analysis parameter settings, we selected a *p*‐value less than .05 to ensure statistical significance and limited the number of output results to 20, which helped us focus on the most important and relevant biological functions and pathways. With these settings, we successfully screened out the biological functions and pathways most relevant to treating hyperlipidemia by *G. pentaphyllum*. To visually present the enrichment analysis results, we used R software (version 4.2.1) to generate a bar graph illustrating the enrichment levels and importance of each function and pathway. These analytical results give us a deeper insight into how *G. pentaphyllum* affects hyperlipidemia and reveal its potential mechanisms (Lu et al., 2020).

### 2.5 Molecular docking

Molecular docking experiments were conducted to investigate the binding mode and affinity of active compounds in *G. pentaphyllum* and their relevant targets. Firstly, the core components of *G. pentaphyllum* were downloaded from the TCMSP database as mol2 files and imported into AutoDock Tools 1.5.6 for processing. The PDBQT format was chosen to save these files to ensure the accuracy of the docking experiments, as it provides a better description of the molecular structure and charge distribution. Subsequently, the 3D structure files of the relevant target proteins were obtained from the Protein Data Bank (https://www.rcsb.org). PyMOL software was employed to remove water molecules and unnecessary inactive ligands from the proteins to improve docking accuracy. The processed protein files were also imported into AutoDock Tools 1.5.6 and subjected to hydrogenation and charge processing before being saved in PDBQT format. Before the docking experiments, various parameters were carefully set to ensure the best docking results. Using AutoDock Vina for semi‐flexible docking, optimal binding conformations between ligands and receptors were obtained. To further evaluate and analyze the docking results, visualizations were performed using PyMOL software. Notably, lower binding energy between ligands and receptors indicates a more stable binding and a higher likelihood of interaction, providing strong evidence for the interaction between active components of *G. pentaphyllum* and their targets (Pinzi & Rastelli, 2019).

### 2.6 Cellular experiments

To investigate the therapeutic effect o*f G. pentaphyllum* on hyperlipidemia at the cellular level, we conducted related cellular experiments using HepG2 cells. First, HepG2 cells were seeded in a 24‐well plate and cultured in a serum‐free medium for 12 h to synchronize the cell cycle after cell adherence. After washing twice with PBS buffer, the cells were cultured in a 10% serum medium. HepG2 cells were treated with 100 μg/mL LDL for 24 h to establish a lipid‐loading model. Subsequently, HepG2 cells were divided into five treatment groups: a normal control group, lipid‐loading model group, and low, medium, and high‐dose intervention groups of *G. pentaphyllum*. In the lipid‐loading model group, cells were only treated with 100 μg/mL LDL for 24 h. In the low, medium, and high dose groups of *G. pentaphyllum*, cells were first pre‐treated with *G. pentaphyllum* total saponins at concentrations of 100, 200, and 400 μg/L, respectively, for 6 h, followed by treatment with 100 μg/mL LDL for 24 h. After the treatments above, the cells were collected, and protein extraction was performed. Using the Western blot technique, we assessed the expression of key proteins, including LOX1, PI3K, AKT, and eNOS, to evaluate the influence of *G. pentaphyllum* on these key proteins and its potential mechanism in the treatment of hyperlipidemia (Taylor & Posch, 2014).

Furthermore, we assessed the protein expression of LOX1, PI3K, AKT, and eNOS by key components of *G. pentaphyllum*, namely rhamnazin, isofucosterol, and quercetin, using a HepG2 cell model. Through preliminary experiments, concentrations of rhamnazin, isofucosterol, and quercetin were determined at 50 μg/mL each. The experimental procedures for these compounds were conducted in a manner consistent with the aforementioned *G. pentaphyllum* cell experiments.

### 2.7 Western blot

The bacterial strain was lysed, and total protein was extracted using a RIPA (Radioimmunoprecipitation Assay) lysis buffer containing 1% PMSF (phenylmethanesulfonyl fluoride) (Catalog Number: P0013B, Supplier: Beyotime, Shanghai, China). Protein extraction was performed according to the instructions provided. The cleared lysate samples were assessed for total protein concentration using the BCA Assay Kit (Catalog Number: P0011, Supplier: Beyotime, Shanghai, China) and adjusted to 1 μg/μL. Each sample was prepared in 100 μL volume, boiled at 100°C for 10 min to denature the proteins, and stored at −80°C until further use. SDS‐PAGE (sodium dodecyl sulfate‐polyacrylamide gel electrophoresis) gels with concentrations of 8%–12% were prepared based on the size of the target protein. Fifty micrograms of protein samples were loaded into each lane using a micropipette. The proteins were separated by electrophoresis at a constant voltage of 80–120 V for 2 h. The gel was then subjected to wet transfer at a constant current of 250 mA for 90 min to transfer the proteins onto a PVDF (polyvinylidene fluoride) membrane (Catalog Number: 1620177, Supplier: Bio‐Rad, USA).

The membrane was blocked with 1xTBST (Tris‐buffered saline with Tween 20) solution containing 5% skim milk at room temperature for 1 h, followed by three washes with 1xTBST washing solution for 10 min each. Next, the membrane was incubated with the primary antibody (Table 1) overnight at 4°C. After washing three times with 1xTBST solution for 10 min each at room temperature, the membrane was washed three times with 1xTBST solution for 5 min each. The membrane was then incubated with HRP‐conjugated goat anti‐rabbit IgG secondary antibody (Antibody Catalog Number: ab6721, Supplier: Abcam, Cambridge, UK; dilution ratio: 1:5000) or HRP‐conjugated goat anti‐mouse IgG secondary antibody (Antibody Catalog Number: ab205719, Supplier: Abcam, Cambridge, UK, Dilution ratio: 1:5000) at room temperature for 1 h. Subsequently, the membrane was washed thrice with 1xTBST buffer for 5 min each at room temperature. The membrane was submerged in ECL (Enhanced Chemiluminescence) reaction solution (Catalog Number: 1705062, Supplier: Bio‐Rad, USA) and incubated at room temperature for 1 min. The liquid was then removed, the membrane covered with plastic wrap, and band exposure imaging was performed using the GE Image Quant LAS 4000C gel imager. β‐Actin was used as an internal control, and the relative expression levels of proteins were determined by the ratio of each band intensity to β‐actin intensity. Protein expression levels were evaluated using reference bands (Wang et al., 2018; Zalucki et al., 2020). Each experiment was conducted in triplicate.

**TABLE 1:** Western blot antibody information.

Target name	Manufacturer	Item number	Dilution ratio
LOX1	Abcam	ab214427	1:1000
PI3K	Abcam	ab302958	1:1000
AKT	Abcam	ab8805	1:500
eNOS	Abcam	ab252439	1:1000
β‐Actin	Abcam	ab6276	1:5000

### 2.8 Statistical analysis

Statistical methods played a crucial role in this research, ensuring the reliability and validity of the conclusions. Firstly, when screening the active compounds and their respective targets in *G. pentaphyllum*, we established specific selection criteria based on oral bioavailability (OB) and drug‐likeness (DL) standards, which required the use of descriptive statistics to determine their distribution. Furthermore, we employed a set theory approach in the intersection analysis between the target proteins of *G. pentaphyllum*'s active compounds and those associated with hyperlipidemia. For the enrichment analysis of GO functions and KEGG pathways, we utilized hypergeometric distribution tests to determine whether a specific function or pathway was significantly enriched while controlling the false discovery rate using adjusted *p*‐values (e.g., Benjamini & Hochberg method). In the cell experiment section, multiple comparisons were conducted, necessitating the use of analysis of variance (ANOVA) or Kruskal–Wallis *H* test (for non‐normally distributed data), followed by post‐hoc tests (e.g., Tukey's HSD) to identify specific intergroup differences. For Western blot experimental data, paired or independent t‐tests might be performed to compare differences between two groups. Throughout all statistical analyses, *p*‐values less than .05 were considered statistically significant. All statistical analyses were conducted using R software (version 4.2.1) and GraphPad Prism software.

## 3 RESULTS

### 3.1 The potential mechanisms of action of active components of *G. pentaphyllum* against hyperlipidemia

**FIGURE 1:**
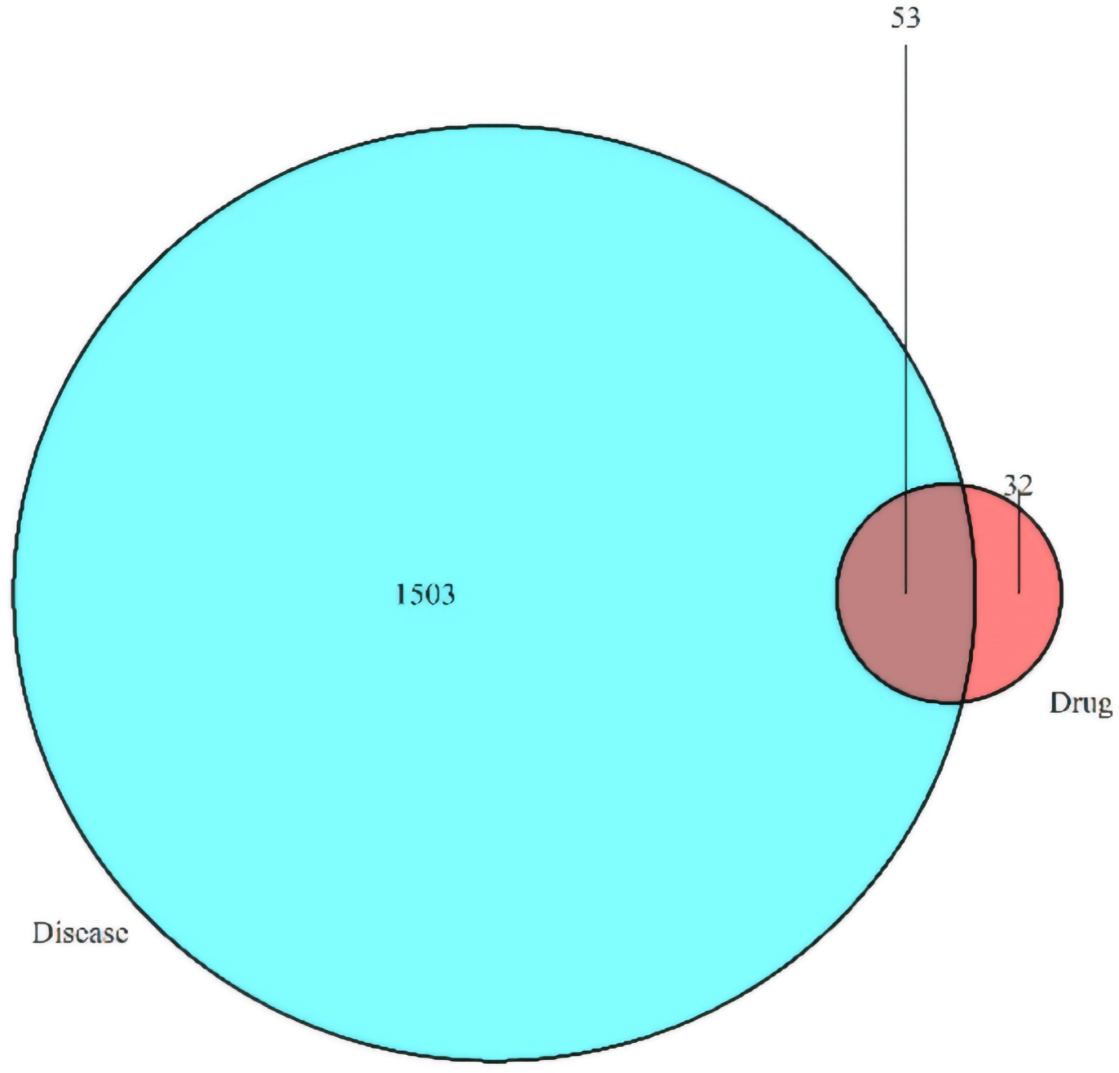
Distribution of 53 shared targets between *Gynostemma pentaphyllum* and hyperlipidemia.

This study investigated the active components and their target actions of *G. pentaphyllum* using network pharmacology methods. The results showed that *G. pentaphyllum* has 85 target actions, while hyperlipidemia has 1556 related targets. Of particular interest are 53 common targets between the two, which indicate that *G. pentaphyllum* may exert its therapeutic effects on hyperlipidemia through these shared targets (Figure 1). Further analysis revealed that *G. pentaphyllum* contains 24 active components, all of which may positively affect treating hyperlipidemia (Table 2). These findings provide a new theoretical basis for applying *G. pentaphyllum* to treating hyperlipidemia and reveal its potential mechanisms of action.

**TABLE 2:** 24 active components in *Gynostemma pentaphyllum* (Jiaogulan) potentially related to the treatment of hyperlipidemia and their proposed mechanisms of action.

MOL ID	Name	MW	OB%	DL
MOL000338	3′‐methyleriodictyol	302.3	51.61	0.27
MOL000351	Rhamnazin	330.31	47.14	0.34
MOL000359	Sitosterol	414.79	36.91	0.75
MOL004350	Ruvoside_qt	390.57	36.12	0.76
MOL004355	Spinasterol	412.77	42.98	0.76
MOL005438	Campesterol	400.76	37.58	0.71
MOL005440	Isofucosterol	412.77	43.78	0.76
MOL007475	Ginsenoside f2	785.14	36.43	0.25
MOL000953	CLR	386.73	37.87	0.68
MOL000098	Quercetin	302.25	46.43	0.28
MOL009855	(24S)‐Ethylcholesta‐5,22,25‐trans‐3beta‐ol	410.75	46.91	0.76
MOL009867	4α,14α‐dimethyl‐5α‐ergosta‐7,9(11),24(28)‐trien‐3β‐ol	424.78	46.29	0.76
MOL009877	Cucurbita‐5,24‐dienol	426.8	44.02	0.74
MOL009878	Cyclobuxine	386.69	84.48	0.7
MOL009888	Gypenoside XXXVI_qt	458.8	37.85	0.78
MOL009928	Gypenoside LXXIV	801.14	34.21	0.24
MOL009929	Gypenoside LXXIX	785.14	37.75	0.25
MOL009938	Gypenoside XII	785.14	36.43	0.25
MOL009943	Gypenoside XL	799.12	30.89	0.21
MOL009969	Gypenoside XXXV_qt	444.77	37.73	0.78
MOL009971	Gypenoside XXVII_qt	418.73	30.21	0.74
MOL009973	Gypenoside XXVIII_qt	416.71	32.08	0.74
MOL009976	Gypenoside XXXII	787.11	34.24	0.25
MOL009986	Gypentonoside A_qt	472.78	36.13	0.8

### 3.2 Network analysis of core active components and key targets

**FIGURE 2:**
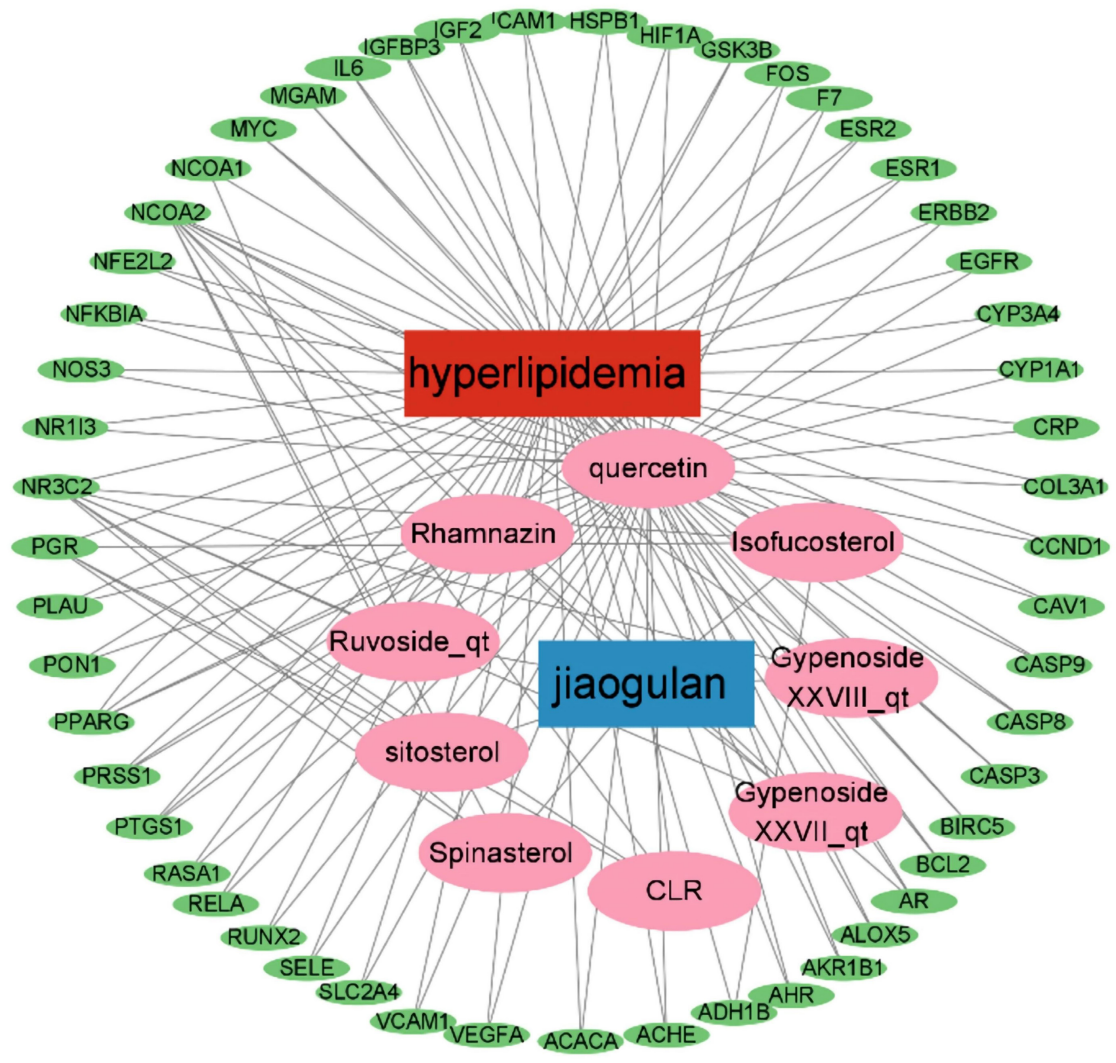
Interaction network of core active components from *Gynostemma pentaphyllum* (Jiaogulan) and key targets in the treatment of hyperlipidemia. *Note*: Network graph of active components and targets (green = targets, pink = active components).

In this study, we investigated the interaction between the components of *G. pentaphyllum* and their targets related to hyperlipidemia using an active ingredient‐target network analysis. By screening and analyzing the core nodes of this network, we have identified several key active components and targets (Figure 2). Specifically, rhamnazin, isofucosterol, and quercetin all have a higher centrality in the network compared to the average value, suggesting their potential core role in treating hyperlipidemia by *G. pentaphyllum*. Additionally, we have identified several critical targets, such as NCOA2, NR3C2, PGR, and PPARG, which also exhibit higher or equal centrality compared to the average value in the network, indicating their potential as major targets for the action of active components of *G. pentaphyllum*. These findings further deepen our understanding of the potential mechanisms behind treating hyperlipidemia with *G. pentaphyllum*.

### 3.3 Analysis of key targets in the PPI network

**FIGURE 3:**
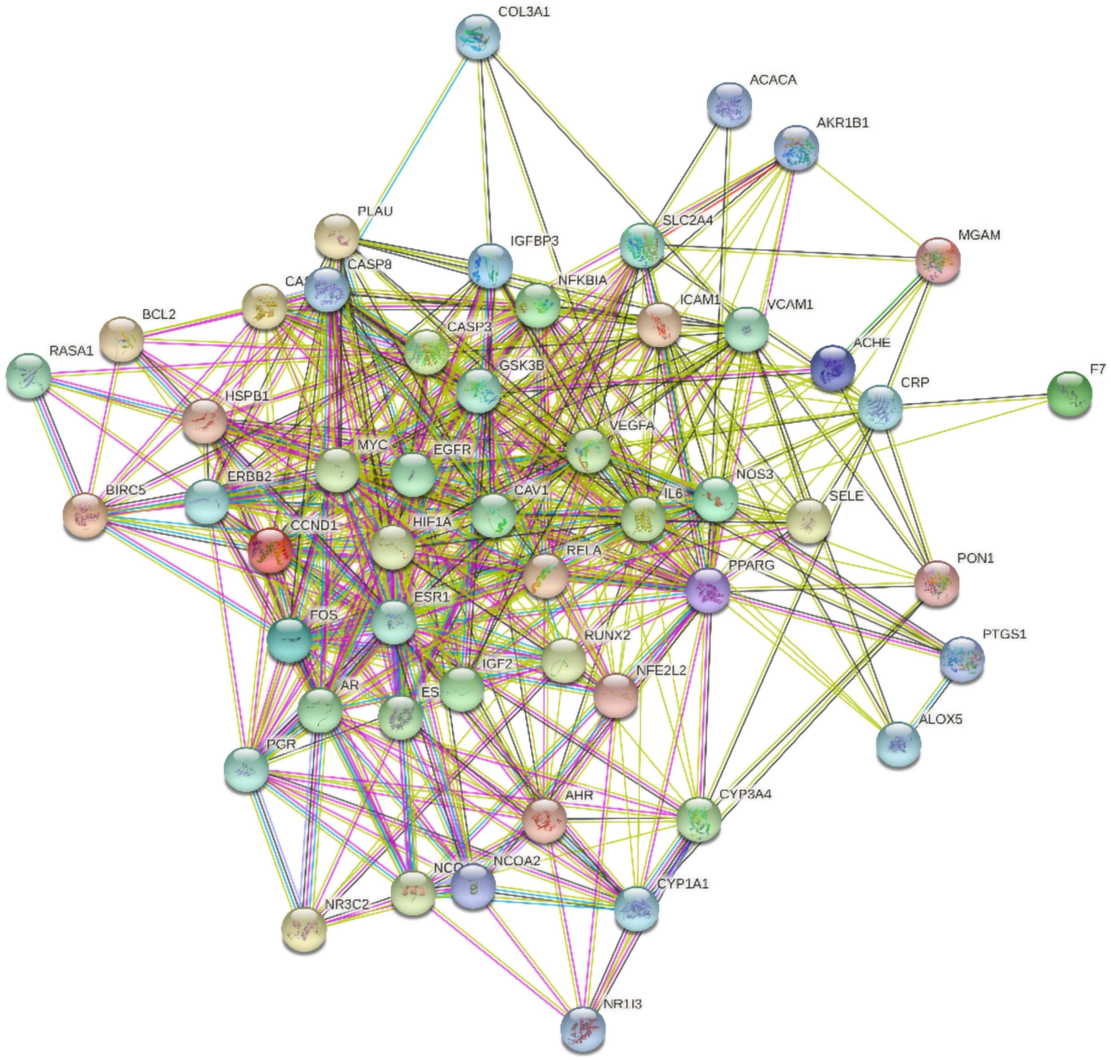
PPI network of common target points for *Gynostemma pentaphyllum* in treating hyperlipidemia.

**FIGURE 4:**
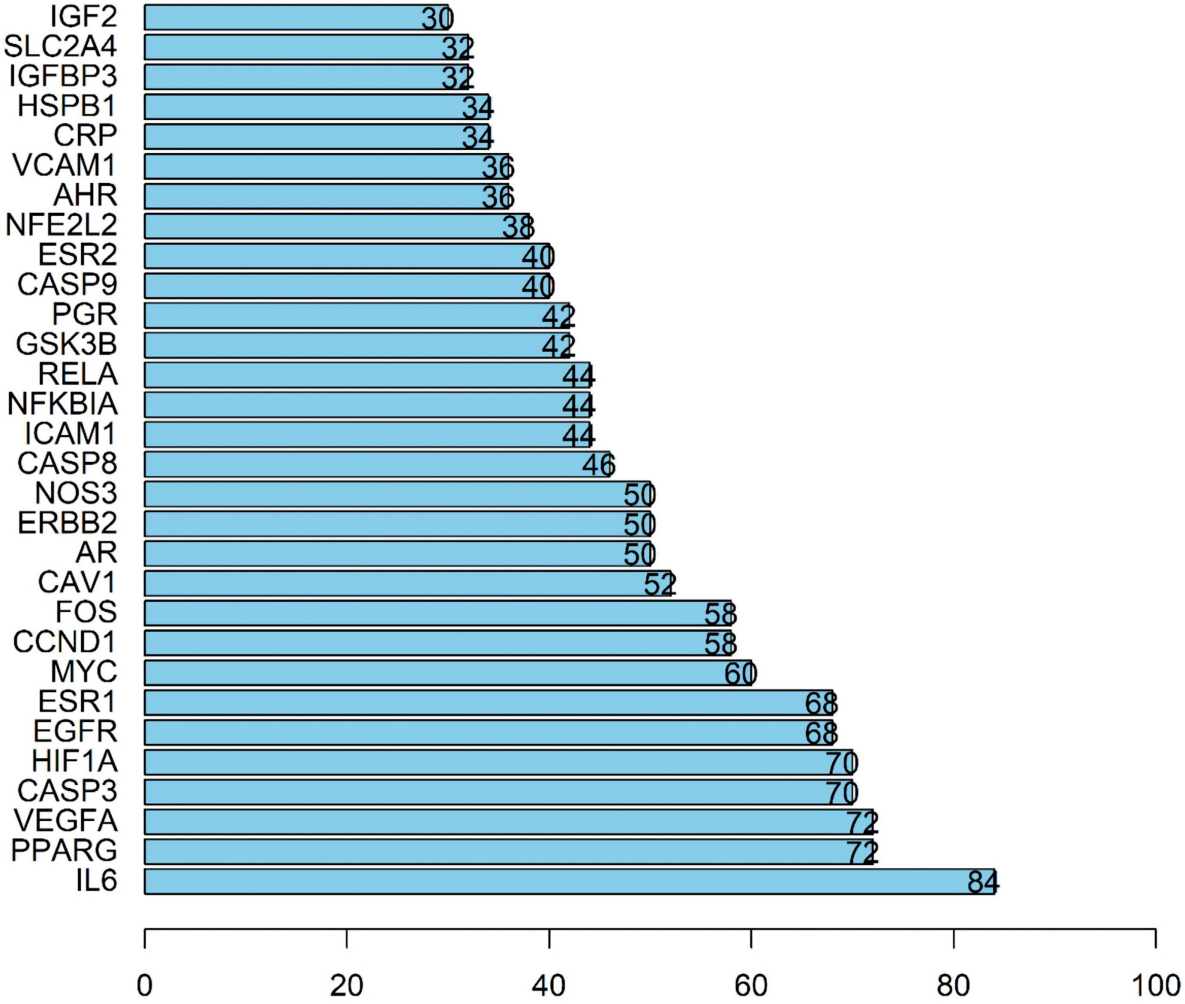
Sequence of targets in the PPI network.

In this study, we further explored the potential interacting relationships of the target proteins of *G. pentaphyllum* in the PPI network. After analysis, the target PPI network comprised 51 nodes and 245 edges (Figure 3). To better understand the importance and centrality of these targets in the network, we ranked them based on their degrees and created a graphical representation of the top 20 key nodes with the highest degrees. Notably, eight nodes, including IL6, PPARG, VEGFA, CASP3, HIF1A, EGFR, ESR1, and MYC, had degrees equal to or exceeding 60, as shown in Figure 4. These highly connected nodes may serve as crucial therapeutic targets for *G. pentaphyllum* in treating hyperlipidemia, with their centrality in the network highlighting their importance in the development and progression of the disease.

### 3.4 Enrichment analysis of biological functions and pathways targeting *G. pentaphyllum*

**FIGURE 5:**
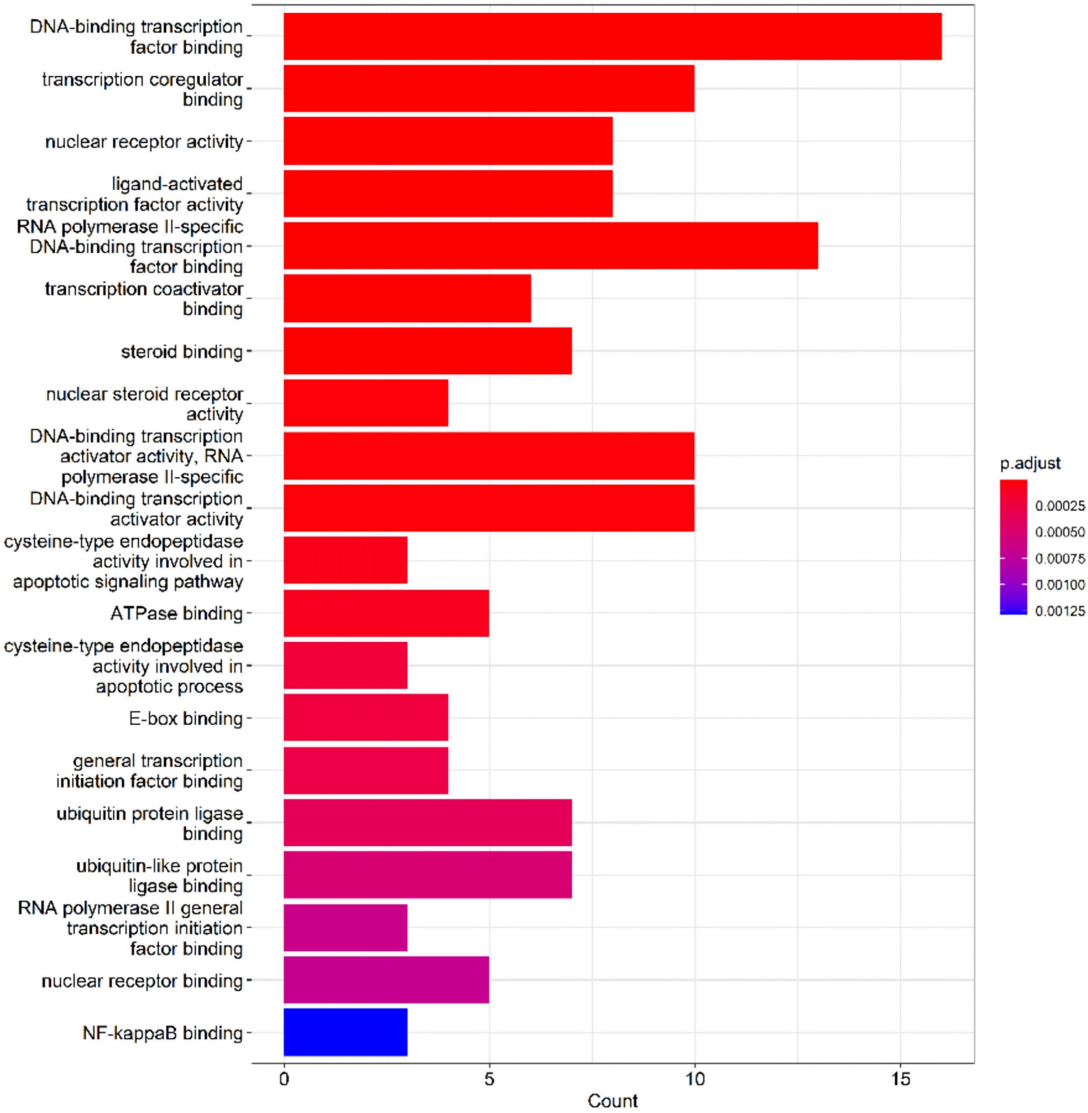
GO functional enrichment analysis.

**FIGURE 6:**
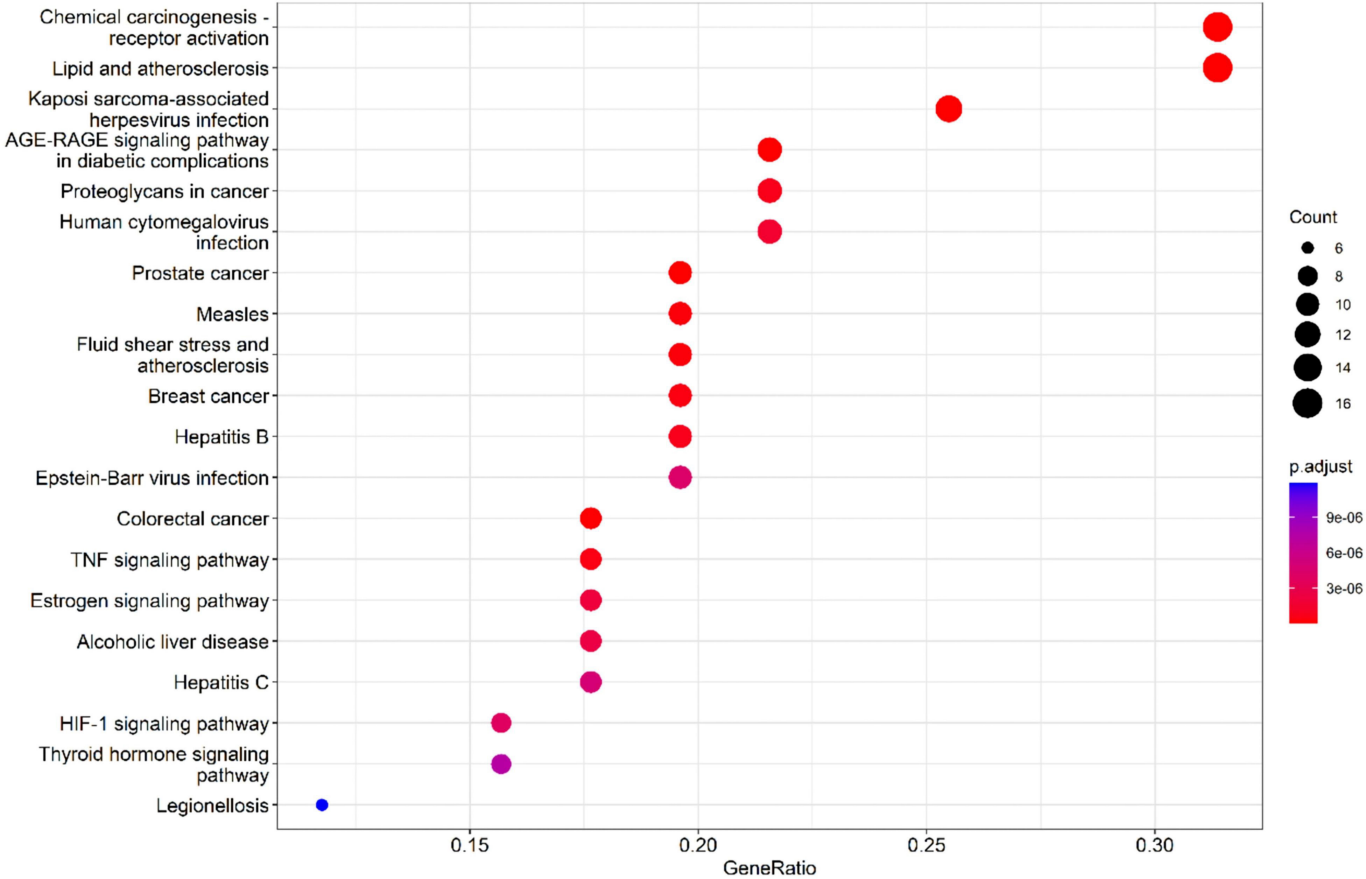
KEGG pathway enrichment analysis.

To further investigate the potential mechanisms of *G. pentaphyllum* in the treatment of hyperlipidemia, we conducted GO function and KEGG pathway enrichment analyses on its target proteins. The results revealed that *G. pentaphyllum* may exert its therapeutic effects by modulating multiple biological functions, particularly those associated with DNA‐binding transcription factor complex and RNA polymerase II‐specific DNA‐binding transcription factor complex, as depicted in Figure 5. Moreover, the subsequent KEGG pathway enrichment analysis identified several major pathways related to the treatment of hyperlipidemia, including lipid metabolism and atherosclerosis, fluid shear stress and atherosclerosis, the TNF signaling pathway, the estrogen signaling pathway, and the PI3K‐Akt signaling pathway, as shown in Figure 6. These pathways could be crucial mechanisms through which *G. pentaphyllum* exerts its therapeutic effects on hyperlipidemia.

### 3.5 Molecular docking analysis of *G. pentaphyllum*'s active ingredients and their corresponding target proteins

**FIGURE 7:**
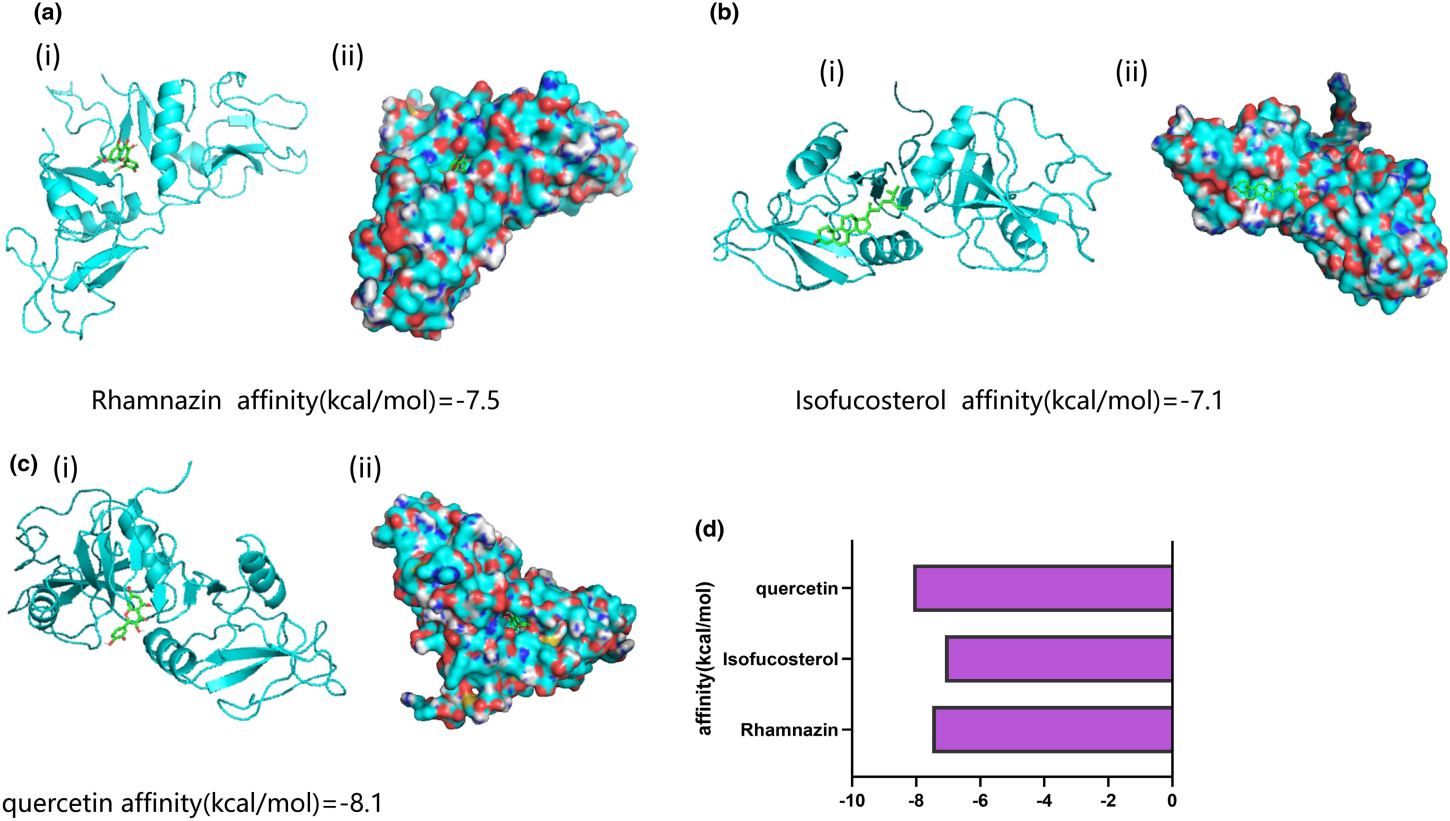
Molecular docking analysis of active components from *Gynostemma pentaphyllum* and their target proteins. *Note*: (a) Docking of Rhamnazin with target protein LOX1; (b) Docking of Isofucosterol with target protein LOX1; (c) Docking of quercetin with target protein LOX1; (d) Histogram of the binding energy of various active components from *Gynostemma pentaphyllum* with target protein LOX1.

A comprehensive molecular docking experiment was conducted to gain deeper insights into the interaction mechanisms between the active ingredients of *G. pentaphyllum* and their potential target proteins (Figure 7). Through the semi‐flexible docking analysis using AutoDock Vina, we successfully obtained the optimal binding conformations of the active ingredients Rhamnazin, Isofucosterol, and quercetin with the target protein LOX1 (Figure 7a–c). Visual analysis using PyMOL software revealed that these active ingredients typically bind to the protein's active pocket or other functionally important regions, implying their potentially significant impact on its function. More importantly, we observed that the binding energies between the ligands and the receptors were predominantly low (Figure 7d), indicating stable interactions between the active ingredients of *G. pentaphyllum* and their target proteins. These low binding energies provide compelling evidence for the high binding affinity between *G. pentaphyllum'*s active ingredients and their targets, which may be a crucial factor contributing to the pharmacological effects demonstrated by *G. pentaphyllum*. Furthermore, the docking results unveiled the key amino acid residues and interaction types between these active ingredients and their targets, providing valuable information to further understand their mechanism of action.

### 3.6 Regulation of the LOX1‐PI3K‐AKT‐eNOS pathway by *G. pentaphyllum* in a high‐fat model of HepG2 cells

To investigate how *G. pentaphyllum* intervenes and affects the relevant pathways of hyperlipidemia, we selected the LOX1‐PI3K‐AKT‐eNOS pathway from the journal Lipid and Atherosclerosis for cellular experiments. Firstly, we successfully established a high‐fat model in HepG2 cells using LDL at 100 μg/mL. To ensure the safety of *G. pentaphyllum*, we preliminarily screened the non‐toxic dosage range using the MTT method.

In the experiment, HepG2 cells were subjected to 6 h of group intervention and divided into five groups: normal control group, lipid‐loaded model group, low‐dose *G. pentaphyllum* group, medium‐dose *G. pentaphyllum* group, and high‐dose *G. pentaphyllum* group. Within 24 h, these cells were treated with 100 μg/mL of LDL. Using the Western blot technique, we quantitatively measured the expressions of LOX1, PI3K, AKT, and eNOS, which are four key proteins.

**FIGURE 8:**
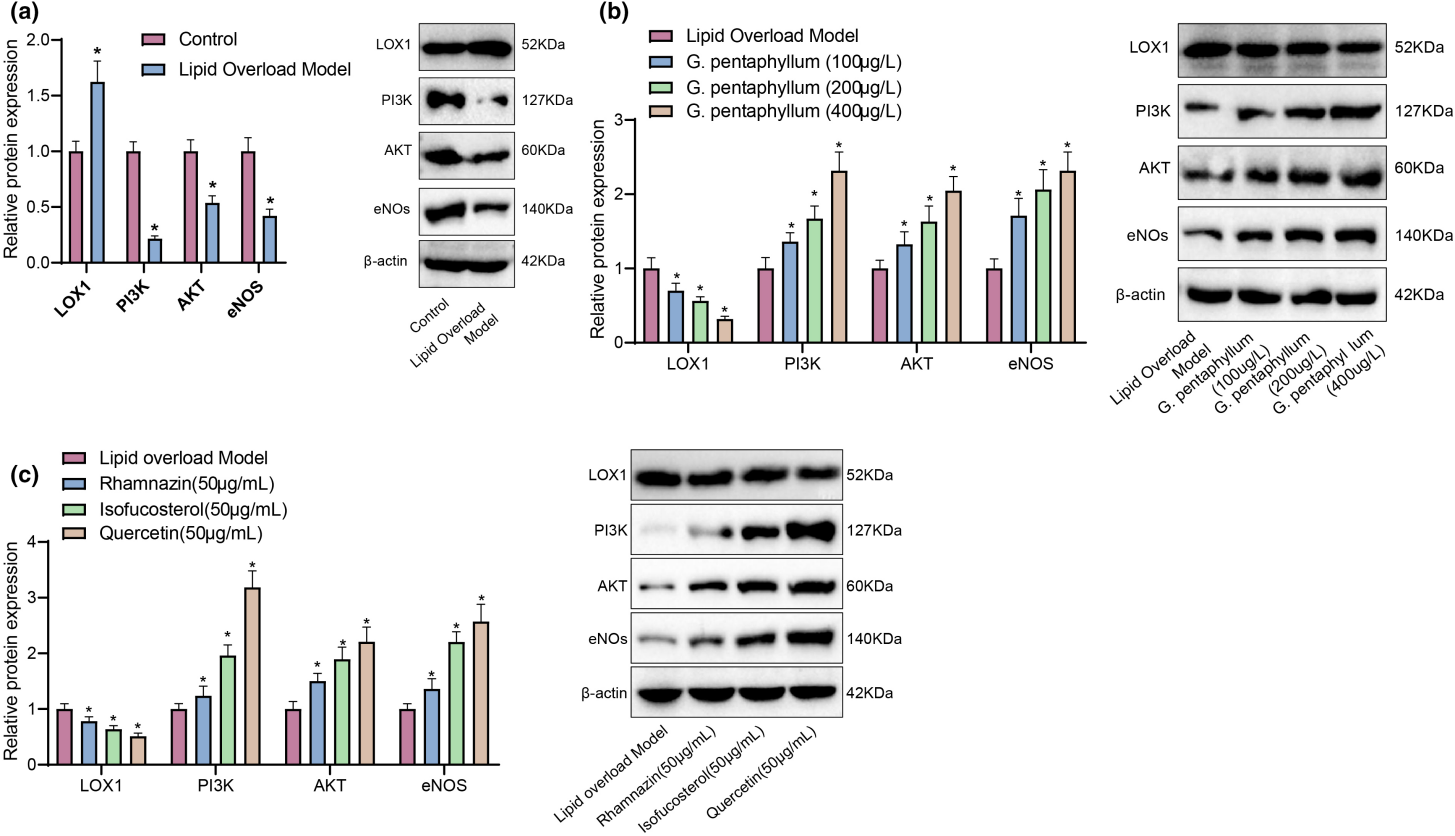
Effects of *Gynostemma pentaphyllum* on LOX1, PI3K, AKT, and eNOS protein expression. *Note*: (a) Western Blot analysis of changes in expression of key proteins LOX1, PI3K, AKT, and eNOS compared to the control group under the lipid‐loading model; (b) Western blot analysis of the regulatory effects of different doses of *Gynostemma pentaphyllum* on LOX1, PI3K, AKT, and eNOS protein expression; (c) regulatory effects of compounds rhamnazin, isofucosterol, and quercetin on the protein expression of LOX1, PI3K, AKT, and eNOS. *A significant difference between the two groups (*p* < .05); cell experiments were repeated three times.

The results showed that, compared to the blank control group, the protein expression of LOX1 in the lipid‐loaded model group significantly increased, while the expressions of PI3K, Akt, and eNOS were significantly downregulated (Figure 8a). Of note, when cells were exposed to different concentrations of *G. pentaphyllum*, the expressions of PI3K, Akt, and eNOS were significantly enhanced in the low, medium, and high‐dose groups of *G. pentaphyllum* compared to the model group, while the protein expression of LOX1 was significantly suppressed (Figure 8b). The main compounds in *G. pentaphyllum*, namely rhamnazin, isofucosterol, and quercetin, have shown the ability to enhance the protein expression of the LOX1‐PI3K‐AKT‐eNOS pathway. These findings align with the positive outcomes observed with *G. pentaphyllum*. Specifically, quercetin exhibited the most pronounced enhancement effect, followed by isofucosterol (Figure 8c). These results provide strong evidence that *G. pentaphyllum* can effectively regulate the protein expression of the LOX1‐PI3K‐AKT‐eNOS pathway, offering a new strategy for treating hyperlipidemia.

## 4 DISCUSSION

Hyperlipidemia has become one of the most common chronic diseases in modern society, posing a significant threat to people's health and lives (Jia et al., 2021; Poornima et al., 2022; Su et al., 2022). With lifestyle changes, the prevalence of hyperlipidemia has been increasing year by year, presenting a huge challenge to public health. In traditional Chinese medicine, *G. pentaphyllum* is well known for its dual effects of health promotion and disease treatment (Ji et al., 2018; Li et al., 2019; Su et al., 2021). Over 230 compounds have been isolated from *G. pentaphyllum*, with the majority (189) being saponins, also known as *G. pentaphyllum* saponins (Li et al., 2016). Although the results indicate the potential of *G. pentaphyllum* and *G. pentaphyllum* saponins in anti‐obesity and cholesterol‐lowering effects (Shaito et al., 2020; Xie et al., 2022), their wide clinical application has yet to be realized due to their complex composition and multiple targets.

Through in‐depth analysis of the chemical constituents of *G. pentaphyllum*, we have successfully screened multiple compounds with potential pharmacological activities. These compounds may have close associations with the therapeutic mechanisms of hyperlipidemia. Further experimental studies have demonstrated that these active ingredients from *G. pentaphyllum* can significantly influence multiple biological pathways associated with hyperlipidemia, providing a theoretical basis for the pharmacological effects of *G. pentaphyllum*.

By constructing an active ingredient‐target interaction network, we have visually presented the relationships between the active ingredients of *G. pentaphyllum a*nd their target actions. These key targets play critical roles in the pathogenesis of hyperlipidemia. Further experimental validation has shown that the active ingredients of *G. pentaphyllum* can significantly regulate the activities of these targets, providing strong support for the treatment of hyperlipidemia with *G. pentaphyllum*.

The PPI network has provided us with a valuable tool to explore the potential target actions of *G. pentaphyllum* and their interactions (Li et al., 2022). We have found that several key targets of *G. pentaphyllum* occupy central positions in the PPI network, indicating their close relationship with the pathogenesis of hyperlipidemia. It further confirms the therapeutic potential of *G. pentaphyllum* for hyperlipidemia.

We conducted functional and pathway enrichment analyses of the target actions of *G. pentaphyllum*, revealing that it may exert its therapeutic effects by regulating multiple biological pathways related to hyperlipidemia. These pathways play crucial roles in the pathogenesis of hyperlipidemia, and the active ingredients of *G. pentaphyllum* may intervene in these pathways to achieve therapeutic effects for hyperlipidemia. Although we conducted network analysis based on existing databases, the inherent biases caused by these databases are a common issue in network analysis. The integrity and timeliness of databases particularly impact our research results. For instance, delayed data updates and the abundance of potential targets for known active metabolites compared to fewer targets for new metabolites may result in false positives or false negatives. To reduce these biases, continuous improvement of databases and timely information updates should be shared objectives in future network analysis studies.

Through molecular docking experiments, we gained a more intuitive understanding of the interaction mechanisms between the active components of *G. pentaphyllum* and their target receptors. The experimental results demonstrated a high affinity between multiple active components of *G. pentaphyllum* and their targets, providing robust evidence for the pharmacological actions of *G. pentaphyllum*. We validated the therapeutic effects of *G. pentaphyllum* and its primary constituents, rhamnazin, isofucosterol, and quercetin, at the cellular level. The results indicated that *G. pentaphyllum* and these components significantly modulate the protein expression of the LOX1‐PI3K‐AKT‐eNOS pathway, offering strong support for the potential mechanisms of *G. pentaphyllum* in the treatment of hyperlipidemia.

Previous studies have reported that oral administration of a single dose of 5000 mg/kg of *G. pentaphyllum* water extract did not induce acute toxicity in female Sprague–Dawley rats. In subchronic toxicity experiments, feeding Sprague–Dawley rats with *G. pentaphyllum* extract at a dose of 1000 mg/kg/day for 90 days showed relative safety of the extract at the given dose. Research has suggested that the effective component *G. pentaphyllum* saponin may lead to herb‐drug interactions by inhibiting CYP2D6, thereby increasing drug toxicity. Establishing appropriate dosages based on these findings is vital for future animal and clinical experiments involving *G. pentaphyllum*; however, further toxicological investigations are necessary.

This study has certain limitations that need improvement. The exploration of *G. pentaphyllum*'s molecular mechanisms for improving hyperlipidemia was solely conducted at the cellular level. Subsequent validation through animal and human trials is imperative. The journey toward the clinical application of *G. pentaphyllum* for treating hyperlipidemia is challenging, necessitating the dete*G. pentaphyllum*rmination of appropriate dosages through animal and human experiments and considerations for biosafety. Furthermore, the impact of complex physiological processes in vivo on the efficacy of *G. pentaphyllum* in improving hyperlipidemia requires further verification. Additionally, focusing solely on the LOX1‐PI3K‐AKT‐eNOS pathway as the mechanism through which *G. pentaphyllum* improves hyperlipidemia, further validation and analysis of other hyperlipidemia‐related signaling pathways obtained through KEGG analysis are required for a more comprehensive understanding of *G. pentaphyllum*'s molecular mechanisms in treating hyperlipidemia.

**FIGURE 9:**
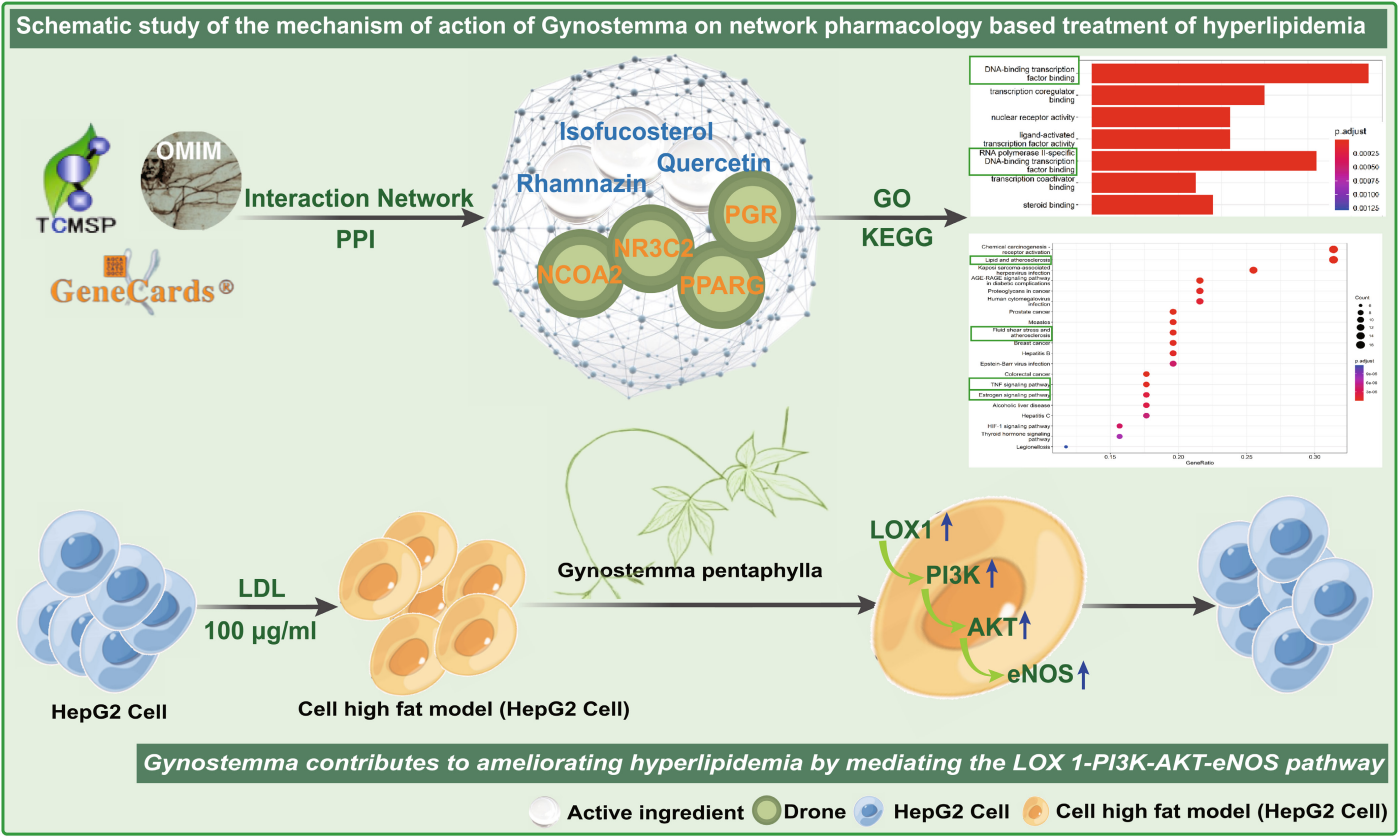
A schematic representation of the mechanism of action of *Gynostemma pentaphyllum* in treating hyperlipidemia based on network pharmacology.

In conclusion, this study has successfully revealed the potential mechanisms of *G. pentaphyllum* in treating hyperlipidemia, providing a strong theoretical foundation for further drug development and clinical applications (Figure 9). Our findings highlight the significant value of *G. pentaphyllum* in traditional and modern medicine.

## AUTHOR CONTRIBUTIONS

**Zhuyang Shen:** Conceptualization (equal); data curation (equal); writing – original draft (equal). **Xin Gao:** Conceptualization (equal); data curation (equal); methodology (equal); resources (equal); visualization (equal). **Dan Huang:** Conceptualization (equal); investigation (equal); project administration (equal); resources (equal); writing – review and editing (equal). **Xiaojin Xu:** Conceptualization (equal); formal analysis (equal); software (equal); validation (equal); writing – review and editing (equal). **Jianping Shen:** Conceptualization (equal); funding acquisition (equal); supervision (equal); writing – review and editing (equal).

## FUNDING INFORMATION

This study was supported by National Natural Science Foundation of China Youth Science Fund Program (82204995) and National Natural Science Foundation of China Program (82374203).

## CONFLICT OF INTEREST STATEMENT

The author declares no conflict of interest.

## Data Availability

The data used in this study are available upon request from the corresponding author, under reasonable circumstances, and in accordance with relevant ethical and privacy considerations.
